# Novel antibody probes for the characterization of endosialin/TEM-1

**DOI:** 10.18632/oncotarget.11018

**Published:** 2016-08-02

**Authors:** Daniel J. O'shannessy, Michael F. Smith, Elizabeth B. Somers, Stephen M. Jackson, Earl Albone, Brian Tomkowicz, Xin Cheng, Young Park, Danielle Fernando, Andrew Milinichik, Brad Kline, Regan Fulton, Pankaj Oberoi, Nicholas C. Nicolaides

**Affiliations:** ^1^ Morphotek, Inc., Exton, PA, USA; ^2^ PhenoPath, Seattle, WA, USA; ^3^ Meso Scale Discovery, Rockville, MD, USA

**Keywords:** endosialin, CD248, TEM-1, tumor microenvironment, sEND

## Abstract

Endosialin (Tumor Endothelial Marker-1 (TEM-1), CD248) is primarily expressed on pericytes of tumor-associated microvasculature, tumor-associated stromal cells and directly on tumors of mesenchymal origin, including sarcoma and melanoma. While the function of endosialin/TEM-1 is incompletely understood, studies have suggested a role in supporting tumor growth and invasion thus making it an attractive therapeutic target. In an effort to further understand its role in cancer, we previously developed a humanized anti-endosialin/TEM-1 monoclonal antibody (mAb), called ontuxizumab (MORAb-004) for testing in preclinical and clinical studies. We herein report on the generation of an extensive panel of recombinant endosialin/TEM-1 protein extracellular domain (ECD) fragments and novel mAbs against ECD motifs. The domain-specific epitopes were mapped against ECD sub-domains to identify those that can detect distinct structural motifs and can be potentially formatted as probes suitable for diagnostic and functional studies. A number of mAbS were shown to cross-react with the murine and human protein, potentially allowing their use in human animal models and corresponding clinical trials. In addition, pairing of several mAbs supported their use in immunoassays that can detect soluble endosialin/TEM-1 (sEND) in the serum of healthy subjects and cancer patients.

## INTRODUCTION

Maintenance of epithelial tissues including those involved in malignant diseases requires interactions with neighboring cells that form tissue stroma. It has been well documented that the formation of solid tumors requires the proliferation of stromal cells to support cancer cell growth, invasion and metastasis [1]. The stromal cell compartment is comprised of a heterogeneous mixture of cells that form blood vessels as well as a microenvironment made up of fibroblasts and leukocytes. Stromal changes at the leading edge of invasive tissues include the appearance of myofibroblasts that share several characteristics with fibroblasts and smooth muscle cells [2]. The coordinated growth and cross talk between stromal cells is key to providing a microenvironment that can support the growth and healthy maintenance of tumor cells. This cross talk is mediated through direct heterotypic cell-cell contacts as well as secreted molecules comprising growth factors, cytokines, chemokines, extracellular matrix (ECM) proteins, proteinases, proteinase inhibitors and glycolipid moieties [3, 4]. Experimental animal models have demonstrated that tumor invasion is stimulated by stromal microenvironments similar to those present in wound healing [5]. This observation suggests that growth factors implicated in wound healing such as transforming growth factor-β (TGFβ) and platelet-derived growth factor (PDGF) may also play a role in changing the stromal host compartment in cancer [6]. In both wound healing and tumorigenesis, the fibroblast to myofibroblast transition marks the stromal alteration that leads to the biological functions needed to support the lesion and perturbation of this process using angiogenic inhibitors, such as the anti-VEGF antibody bevacizumab, suppress the natural wound healing process.

The stromal microenvironment is also important for supplying blood and nutrients to tumor cells *via* angiogenesis, which is also critical for physiological tissue growth, wound healing and embryo development [7]. As part of the angiogenic process, fibroblasts have been found to serve a vital role in secreting ECM proteins that are required for modeling and stabilizing the budding edge and vascular network of *de novo* blood vessels [8]. These proteins constitute a structural scaffold for proliferating endothelial and tumor tissues and, more importantly, provide support for the attachment of tumor cells. These vascular structures are comprised of pericytes whose function is the stability of endothelial cell-cell assembly and vessel sprouting that in turn provides support for the vessel lumen and blood flow to the tumor microenvironment [9]. In light of the critical relationship of tumor and stromal cells, anti-cancer strategies aimed at disrupting the tumor stromal cell compartment, including suppression of angiogenesis are being pursued [10].

Several approaches have been used in an attempt to identify cell surface markers on tumor stromal cells to better define their subtypes as well as for potential targeted therapy. Endosialin, also called Tumor Endothelial Marker-1 (TEM-1) or CD248 is one of several proteins that have been identified to be localized to the tumor stromal compartment [11, 12]. The protein was first discovered using a whole cell immunization approach whereby human embryonic fibroblasts, which share many characteristics with stromal cell fibroblasts were used to immunize immuno-competent mice. These efforts led to the identification of the monoclonal antibody FB5 that was able to recognize an antigen present on tumor stromal cells and malignant cells of mesenchymal origin that was named endosialin [13]. An independent method was also used to identify cell surface markers on primary tumor endothelium *via* Serial Analysis of Gene Expression (SAGE). This research identified the TEM-1 gene product that subsequently was determined to be the FB5 antigen [14]. Further examinations of gene expression patterns in normal and neoplastic tissue have found a consistent up-regulation of endosialin/TEM-1 expression in tumor neovessels. These include enhanced expression of endosialin/TEM-1 in stroma of human colorectal cancer [10, 15], breast cancer [16, 17], histiocytomas [18] and expression directly on tumor cells of mesenchymal origin including sarcoma [19, 20] and melanoma [21, 22]. Human endosialin/TEM-1 expression has also been reported in highly invasive glioblastoma, anaplastic astrocytomas and metastatic carcinomas [21, 23]. Refined localization studies have delineated endosialin/TEM-1 expression to tumor-associated pericytes and at the leading edge of tumor vessel sprouting while very low levels of endosialin/TEM-1 have been reported in vessels of normal organs [24, 25].

Functional studies have shown that endosialin/TEM-1 knockout (TEM-KO) mice develop normally and exhibit normal wound healing, suggesting that endosialin/TEM-1 is not required for neovascularization during fetal development or wound repair as is the case for normal angiogenesis [26]. When colorectal cancer cells were implanted orthotopically in the abdomin of TEM-KO mice, the lack of endosialin/TEM-1 expression correlated with a drastic reduction in tumor growth, invasion and metastases as compared to parental animals. These results suggest that stromal and/or endothelial-associated cells expressing endosialin/TEM-1 support tumor growth and invasion perhaps *via* the interaction with cellular and ECM proteins within the microenvironment of the tissue of origin.

Based on the important role of stroma in supporting tumor growth and the activity of endosialin/TEM-1 in supporting tumor stromal cell functions, clinical studies using a humanized monoclonal antibody called ontuxizumab (MORAb-004) that can perturb endosialin/TEM-1 biology are currently being conducted to determine the safety and clinical activity in a variety of cancer types [27]. Preclinical studies with ontuxizumab have shown that treatment of tumor-bearing mice resulted in small, dysfunctional vessels in the tumors and that endosialin/TEM-1 expression on neovascular pericytes was decreased due to antibody-mediated internalization [28]. These results are consistent with a report by Naylor et.al. that demonstrated a role for endosialin/TEM-1 in PDGF-mediated angiogenesis in skeletal muscle [29], whereby TEM-1-deficient mice had defects in vessel sprouting also but not splitting nor overall angiogenesis during muscle remodeling. Endosialin/TEM-1 has also been shown to be directly involved in regulating cellular proliferation [30] and in a subset of cells this proliferation appears to involve the PDGFRβ pathway, a pathway reported to be highly associated with tumor stromal cell proliferation and resistance to signal transduction therapies [6, 31].

The extracellular domain of endosialin/TEM-1 shares structural and sequence homology with thrombomodulin (CD141) and C1qRp (CD93) proteins [32, 33]. Three distinct regions of homology have been identified: a C-terminal lectin-like domain (CTLD), a Sushi domain and a three EGF repeat domain. Structural homologies to two receptor proteins suggest that endosialin/TEM-1 may be a cell surface receptor. Molecular and cellular studies have previously found that endosialin/TEM-1 is able to selectively bind to the ECM proteins fibronectin (FN) and collagen types I and IV (Col I, Col IV). Engineered cells expressing endosialin/TEM-1 exhibit enhanced adhesion to FN as well as enhanced migration through tumor matrices containing FN [34] which are suppressed by antibodies targeting endosialin/TEM-1 CTLD. The functional activities of the Sushi and EGF domains remain to be elucidated.

Here we describe the generation of a panel of recombinant endosialin/TEM-1 proteins and novel monoclonal antibodies that are specific to distinct regions of the endosialin/TEM-1 protein. These reagents will aid in further understanding the biology of the endosialin/TEM-1 pathway in normal tissue and in endosialin/TEM-1-positive tumors and tumor associated stroma. Moreover, the combination of these reagents enabled the development of immunoassays that can be used to determine if prognostic or predictive endosialin/TEM-1 serum (sEND) or protein expression profiles exist in patients with various types of cancer.

## RESULTS AND DISCUSSION

### Production and screening of mAbs

In order to better characterize the expression and function of human endosialin/TEM-1, we developed a panel of rat mAbs directed against a recombinant human endosialin/TEM-1 construct comprising the entire ECD of the protein. Fusions resulted in a number of positive hybridoma clones, a subset of which was selected for further characterization.

**Figure 1:**
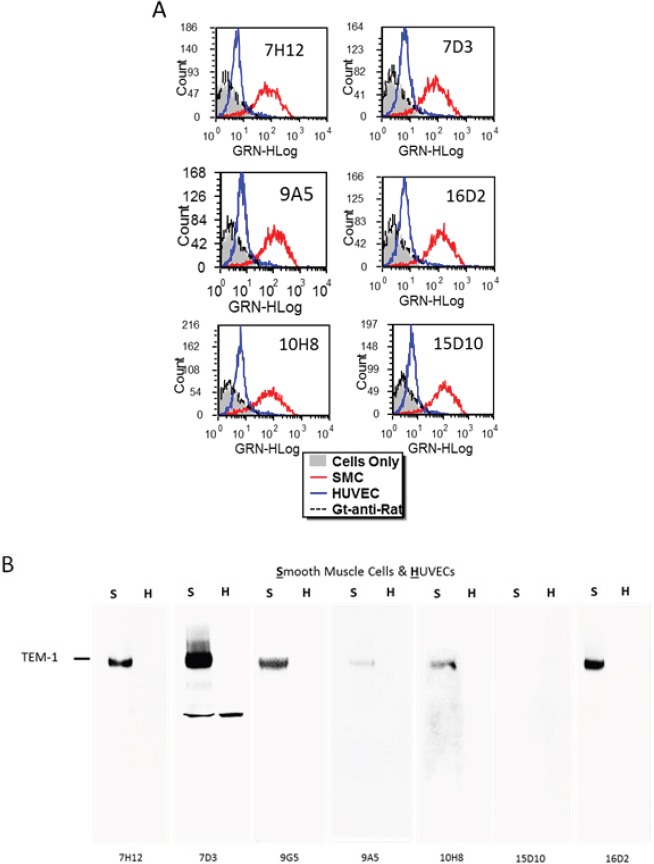
Screening of rat anti-human endosialin/TEM-1 mAbs by FACS (Figure 1a) and western blot (Figure 1b) on aortic smooth muscle cells (S) or human umbilical vein endothelial cells (H)

Antibodies were first analyzed for cell surface binding to endosialin/TEM-1 by FACS using aortic smooth muscle cells (SMCs) (endosialin/TEM-1^+^) and human umbilical vein endothelial cells (HUVECs) (endosialin/TEM-1^−^) (Figure 1A). Results showed that all of the selected mAbs had robust binding to SMCs (blue) in contrast to HUVEC control cells (red). Non-specific isotype control antibody (black) had similar non-binding profiles. In order to further demonstrate specificity, mAbs were next assessed by western blot (WB) using lysates from SMCs (TEM-1^+^) or HUVECs (TEM-1^−^). Results from these analyses showed that only a subset of antibodies were able to detect a ~160 kDa band corresponding to the expected molecular weight of glycosylated endosialin/TEM-1 in SMC while no reactivity was found for any in the HUVEC cell lysates (Figure 1B). Since the SDS-PAGE was done under reducing/denaturing conditions, it was to be expected that not all antibodies would be able to detect endosialin/TEM-1 due to the potential loss of conformational epitopes as demonstrated using mAb-9A5 that reacted robustly by FACS of SMCs, performed under native conditions, but weakly detected endosialin/TEM-1 in denatured WB suggesting that it recognizes a conformational epitope. Similarly, mAb-15D10 did not recognize endosialin/TEM-1 under reduced WB conditions but recognized native endosialin/TEM-1 *via* FACS.

**Figure 2:**
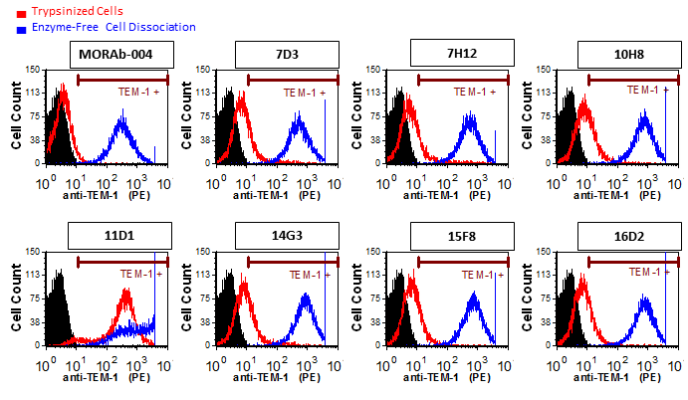
Cell surface expression of endosialin/TEM-1 is trypsin-sensitive FACS analysis of SJSA-1 cells removed from tissue culture flasks with enzyme-free cell dissociation buffer (blue) or trypsin (red). The of endosialin/TEM-1 region thatis detected by mAb-11D1 is trypsin insensitive.

Subsequently, some disparate FACS data led us to evaluate different methods of cell dissociation from tissue culture plates on antibody binding to endosialin/TEM-1. As shown in Figure 2, recognition of the endosialin/TEM-1^+^ SJSA-1 osteosarcoma cell line was markedly affected by the cell dissociation process. With the exception of mAb-11D1, none of the antibodies tested could detect cell surface endosialin/TEM-1 when trypsin was used for disociation. These results indicate that endosialin/TEM-1 contains at least one trypsin-sensitive cleavage site within its extracellular domain. This is supported by protein sequence analysis using the web-based ExPASy peptide cutter tool [35], which predicted the presence of over 30 potential trypsin cleavage sites in the endosialin/TEM-1 ECD. Since mAb-11D1 reacts well with trypsin-treated cells it is likely that it binds to a more membrane proximal epitope relative to the others tested and this was confirmed using polypeptide domain mapping (see below).

### Domain mapping by WB and EIA

The endosialin/TEM-1 ECD is predicted to contain several motifs whose functions are yet to be defined. Immediately following the N-terminal leader sequence is a C-type lectin-like domain (CTLD; aa30-156). Previous studies have demonstrated that this region is important for binding to ECM proteins such as collagen and fibronectin [34]. Juxtaposed to the CTLD is a Sushi domain (SD; aa162-232) which shares homology with proteins involved in complement regulation. Downstream of the Sushi domain are three EGF-like repeats (aa312-351) followed by the heavily glycosylated mucin domain (aa400-687).

**Figure 3:**
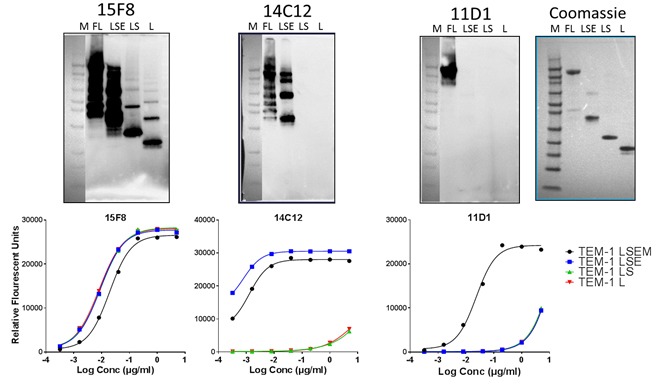
Domain mapping of rat anti-human endosialin/TEM-1 antibodies Top: Non-reducing western blot; Bottom: Direct EIA against immobilized endosialin/TEM-1 fragments. M: MW markers; FL: full length ECD (LSEM: lectin, Sushi, EGF domains, mucin domain); LSE: lectin, Sushi, EGF domains; LS: lectin, Sushi domains; L: lectin domain; Coomassie blue stained SDS-PAGE of fragments shown for comparison.

**Figure 4:**
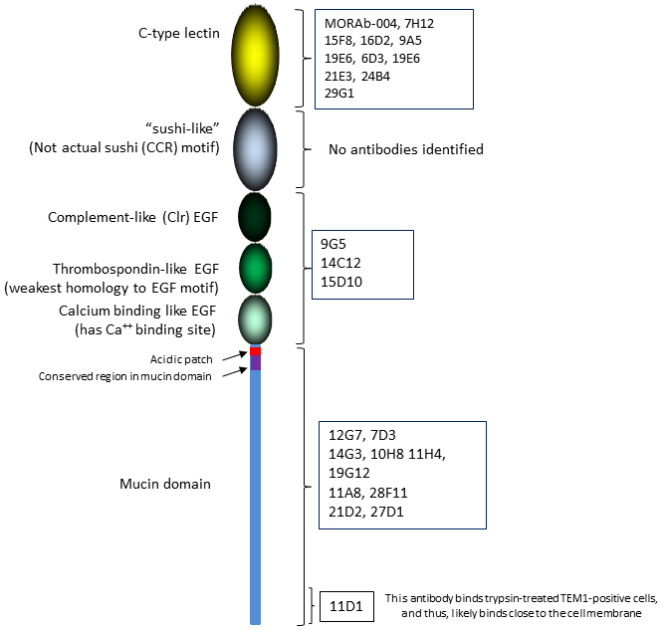
Summary of domain mapping of rat anti-human endosialin/TEM-1 antibodies as determined by western blot and ELISA using extracellular domain fragments

To better delineate the function of the ECD motifs, we constructed a series of endosialin/TEM-1 truncated polypeptides in an effort to identify mAbs with distinct motif binding characteristics. Recombinant proteins corresponding to the entire ECD (LSEM; Lectin-Sushi-EGF-Mucin), Lectin-Sushi-EGF (LSE), Lectin-Sushi (LS), and Lectin only (L) domains were generated and screened with our panel of mAbs *via* WB, under non-reducing conditions and by direct EIA. Figure 3 shows the results for three representative mAbs with distinct domain binding specificities. mAb-15F8 recognized all four endosialin/TEM-1 truncations by both WB and EIA, indicating that its binding epitope resides within the lectin domain. In contrast, mAb-14C12 did not bind to the LS or L fragments but could bind the LSEM and LSE fragments indicating that its binding epitope is localized to the EGF-like domain(s). mAb-11D1 only bound full length LSEM demonstrating that its epitope lies within or close to the C-terminal proximal region of the mucin domain. Coupled with the data obtained by FACS and the results of trypsinization, it is likely this mAb binds in close proximity to the transmembrane portion of endosialin/TEM-1. Results of domain mapping for all mAbs assessed are presented pictorially in Figure 4 and Table 1. With the exception of the Sushi domain, most mAbs were identified to react specifically with each of the distinct domains.

**Table 1:** Summary of rat anti-human endosialin/TEM-1 mAb characteristics

		Domain Mapping						Western Blot			
Clone ID	Isotype	Lectin	Sushi	EGFs	Mucin	ELISA	FACS	reduced	non-reduced	X-reactivity with mouse	KD^5^
7H12	IgG1/k	+				+	+	+	+	weak	0.034 nM
15F8	IgG2a/k	+				+	+	+/−	+	−	0.24 nM
16D2	IgG2a/k	+				+	+		+	−	0.24 nM
9A5	IgG2a/k	+				+	+	+/−	+	−	1.5 nM
19E6	IgG1/k	+				+			+	−	
21E3	IgG1/λ	+				+			+/−	weak	
29G1	IgG1/k	+				+			+	very weak	
24B4	IgG2a/k	+				+			+	−	
6D3	IgG2a/k	+				+			+	strong	
9G5	IgG1/k			+		+	+	+	+	moderate	1.9 nM
15D10	IgG2a/k			+		+	+	+/−	+	strong	20.5 nM
14C12^1^	IgG2a/k			+		+		+	+	−	
12G7	IgG1/k				+	+		+	+/−	−	
7D3	IgG2a/k				+	+	+	+	+/−	weak	
14G3	IgG2a/k				+	+	+	+	+	strong	35 nM
10H8	IgG2a/k				+	+	+	+		−	
11H4	IgG2a/k				+	+		+			
19G12	IgG1/k				+	+		+		−	
11A8^2^					+	+/−		+/−		−	
28F11	IgG2a/k				+			+	+/−	weak	
21D2	IgG2a/k				+	+		+	+/−	−	
27D1^2^					+	+		+/−		moderate	
11D1^3^	IgG2a/k				+	+	+	+		−	
5D11^4^	IgG2a/k				+	+		+	+	−	

1 Binds only full length protein under reducing conditions

2 Reacts weakly in domain mapping western blot and ELISA

3 Binds trypsin-treated TEM-1^+^ cells, thus likely near membrane attachment

4 Very clean high molecular weight western signal, no fragments recognized, possibly near membrane attachment

5 As determined by surface plasmon resonance

### mAb cross-reactivity with mouse endosialin/TEM-1

Few anti-human endosialin/TEM-1 antibodies have been described that can react with the mouse ortholog and potentially be used for studies aimed at exploring the biological function of endosialin/TEM-1 *in vivo*. MacFayden et.al. described antibody p13 in studies examining the regulation of endosialin/TEM-1 expression during mouse development [36] and this antibody has been used for immunohistochemical detection endosialin/TEM-1 in several studies. Biobody-78, a single chain Fv (ScFv) antibody fragment derived from a yeast display library has also been developed as an experimental imaging agent by Zhao, et al. [37] and is cross-reactive with both the human and mouse orthologs. To date, much of our knowledge regarding the role of endosialin/TEM-1 in normal physiology or disease models has been derived from xenograft tumor models in immuno-incompetent mice or from endosialin/TEM-1/CD248 knockout mice [26, 29; 38–41]. Thus, the generation of a well characterized panel of endosialin/TEM-1 mAbs reactive to human and the mouse ortholog, that cover an array of distinct ECD domain specificities serve as useful reagents to further elucidate the biological function(s) of endosialin/TEM-1.

**Figure 5:**
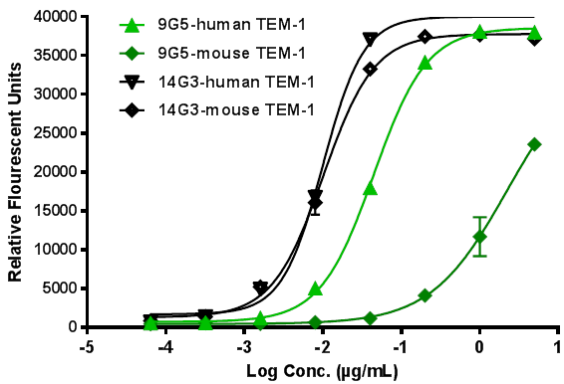
Cross-reactivity of select mAbs with mouse endosialin/TEM-1 EC50 for human endosialin/TEM-1: mAb-14G3=0.01 μg/mL; mAb-9G5=0.044 μg/mL; EC50 for mouse endosialin/TEM-1: mAb-14G3=0.01 μg/mL; mAb-9G5=2.2 μg/mL.

To determine if any of the anti-human endosialin/TEM-1 mAbs described herein cross-react with the murine endosialin/TEM-1 ortholog, mAbs were tested in a direct EIA format using either immobilized mouse or human endosialin/TEM-1 ECD protein fragments. A representative experiment is presented in Figure 5 showing that mAb-14G3, which maps to the mucin domain, was equally reactive to both the human and mouse proteins. In contrast, mAb-9G5, which maps to the EGF-like domain, is weakly cross-reactive with mouse endosialin/TEM-1. In this manner, the reactivity of all mAbs to mouse endosialin/TEM-1 was determined and the data are summarized in Table 1. Importantly, we were able to identify cross-reactive mAbs that bound robustly to the lectin (mAb-6D3), EGF (mAb-15D10) and mucin (mAb-14G3) domains of endosialin/TEM-1. The use of these cross reactive antibodies may provide tools to further characterize the biology of endosialin/TEM-1 in development and disease in addition to uncovering potential roles of the various ECD domains in tumor biology.

### Soluble endosialin/TEM-1 (sEND) is present in serum in both healthy individuals and cancer patients

Many cell surface proteins have been found in systemic circulation, some of which have proven useful for the diagnosis or monitoring of disease progression or as a determinant of therapeutic response. For example, circulating CA-125 and HE4 are routinely measured for the management of ovarian cancer patients [42, 43] while circulating mesothelin is useful in the diagnosis of mesothelioma [44]. Given the lability of endosialin/TEM-1 to trypsin (shown in Figure 2), we hypothesized that it might also be cleaved and detectable as a soluble protein in systemic circulation.

**Figure 6:**
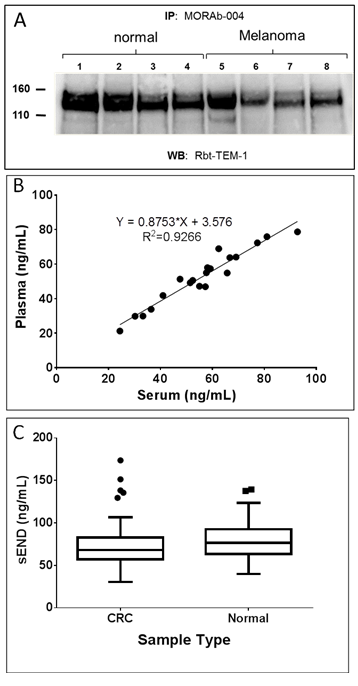
Detection of soluble endosialin/TEM-1 (sEND) in human serum **A.** Endosialin/TEM-1 was immunoprecipitated from serum with MORAb-004, separated by SDS-PAGE, and detected using a rabbit anti-human TEM-1 polyclonal Ab. **B.** Correlation between measurements of sEND in plasma *vs*. serum by ECL method. **C.** Presence of sEND in colorectal cancer and normal serum. Boxes represent the 25^th^ to 75^th^ percentile ranges. Whiskers represent 1.5 times the interquartile range (Tukey method) and solid line is the median. *n* = 117 for colon cancer; *n* = 100 for normal.

To test this hypothesis, we first performed immunoprecipitation experiments using ontuxizumab (MORAb-004) on a number of serum samples derived from healthy individuals and melanoma patients. These studies found that a number of samples contained soluble endosialin/TEM-1 (sEND) species in the ~120-150KDa range representing the expected molecular weight of glycosylated ECD protein (Figure 6A). To our knowledge, this represents the first demonstration that a soluble form of endosialin/TEM-1 exists in the serum of healthy individuals and cancer patients. Similar results were found using independent antibodies to endosialin/TEM-1 that bind to different regions of the ECD than MORAb-004 (not shown), demonstrating specificity of these results. To determine if a quantitative difference in steady state levels of sEND exists between cancer patients and healthy individuals we sought to develop a quantitative sandwich immunoassay for sEND by employing a combination of the novel mAbs described herein.

After pairwise screening of the mAbs described above, a final electrochemiluminescent (ECL) assay was developed that showed good linear sensitivity and specificity for sEND. The assay employed mAb-9G5 (biotinylated) as the capture antibody and mAb-15F8 (ruthenium labeled) as the detector antibody in a solution phase assay format with [9G5-sEND-15F8] complexes being captured onto streptavidin plates and quantitated. The sEND ECL assay had a linear dynamic range of 0-5000 pg/mL, an LLOD of 1.35 pg/mL and an LLOQ of ≈5 pg/mL. In order to identify the most appropriate matrix for measuring sEND levels, 20 matched serum and plasma samples were assayed. As shown in Figure 6B, there was no preferential partitioning of sEND into either plasma or serum and therefore either sample preparation appears suitable for use in this assay. In addition, sEND appears stable through at least 3 freeze/thaw cycles in both serum and plasma (data not shown). Other antibody parings have also detected similar baseline levels of sEND in serum and plasma (not shown).

To explore the potential utility of sEND as a biomarker for clinical assessment, we evaluated levels in serum samples from 117 colorectal cancer patients (CRC; stages III and IV) and 100 healthy control individuals. As shown in Figure 6C, there was no difference in levels of sEND between these two groups. This is somewhat unexpected given the previously reported findings that elevated expression of endosialin/TEM-1 occurs in the tumor stromal compartment, most notably in CRC [10, 15], while limited expression exists in normal adult tissues. Of note are the quite high levels of sEND in serum, in both healthy individuals and cancer patients, with circulating levels ranging from 10 to >100 ng/mL. Endosialin/TEM-1 has been classified as a mesenchymal stem cell (MSC) marker/antigen [45]. MSCs are the precursors of pericytes and stromal fibroblasts and the relatively high endogenous serum levels of sEND may reflect the state of flux of tissue regeneration/remodeling, even in healthy individuals. Furthermore, while little is known about the clearance of sEND in serum, it might be expected to have a significant half-life due to its large molecular mass, possibly in the same order as similarly sized molecules such as immunoglobulins. Given these preliminary data, it is clear additional studies are required to define if any value may exist in monitoring sEND levels for the prognosis of cancer and/or predictive response to therapy.

### Immunohistochemical detection of endosialin/TEM-1 in normal and tumor tissue

**Figure 7:**
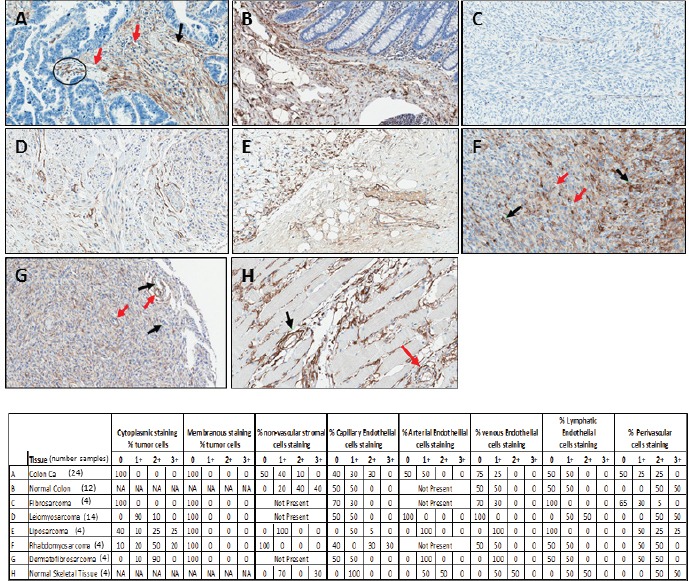
Examples of immunohistochemical detection of endosialin/TEM-1 in a subset of malignant and normal tissues All images are at 20X magnification. Table indicates percentages of target cells staining at the following intensities: 0 (defined as negative), 1+ (defined as weak), 2+ (defined as moderate), or 3+ (defined as strong). The number of samples tested for each tissue is in parentheses and listed in the summary table. **A.** Colorectal cancer (CRC) with absent staining of tumor cells and strong staining of capillary endothelial cells (black circle). There is variable staining of venous (red arrows) and lymphatic endothelium (black arrow). **B.** Normal colon. The capillary endothelium is predominantly negative. Venous and lymphatic endothelial cells show variable weak staining, while perivascular cells and non-vascular stromal cells are strongly stained. **C.** Fibrosarcoma showing no tumor cell staining. Weak staining is seen among capillary and venous endothelial cells. Lymphatic endothelial cells are negative and perivascular cell staining is patchy and weak. **D.** Leiomyosarcoma with weak staining of the tumor cell cytoplasm. Strong perivascular cell staining is seen. Variable staining is seen among vascular endothelial cells. **E.** Liposarcoma showing variable/weak cytoplasmic staining among tumor cells. Non-vascular stromal cells show uniform weak staining. Venous and lymphatic endothelial cells show weak to moderate staining, while capillaries show moderate to strong staining. Perivascular staining is moderate to strong. **F.** Rhabdomyosarcoma showing variable cytoplasmic staining among tumor cells. Capillary endothelial cells show dichotomous staining, negative among approximately ½ (red arrows) and strongly positive in the remainder (black arrows). **G.** Dematofibrosarcoma showing variable moderate cytoplasmic tumor cell staining. Perivascular cells (red arrows) are moderate to strongly stained, while vascular endothelial cells (black arrows) are negative or only faintly stained. **H.** Normal skeletal muscle. The capillary endothelial cells are uniformly weakly stained, and there is variable moderate staining for the endothelial component of arteries (red arrow), veins and lymphatics. Perivascular cells are highlighted with strong staining (black arrows).

The use of the mAb-9G5 for the detection of endosialin/TEM-1 in melanoma by immunohistochemistry [22] and in colorectal cancer by immunofluorescence [15] has been described previously and its expression was found to correlate with mRNA expression patterns. To further explore the expression of endosialin/TEM-1 in normal and tumor tissue, we used mAb-9G5 to characterize endosialin/TEM-1 expression in colorectal cancer, sarcomas and normal colon (Figure 7). Staining was evaluated by a board certified pathologist relative to intensity and cell type and these data are presented for each sample in Figure 7.

In CRC, endosialin/TEM-1 expression was localized to non-vascular stromal cells and capillary endothelial cells, while no staining was observed in the carcinoma component, which is consistent with previous reports [15]. In contrast, in normal colon the capillary endothelial cells were predominantly negative or weak while moderate to strong staining was observed in perivascular and stromal cells.

Endosialin/TEM-1 expression in sarcoma samples was variable with some samples lacking any expression (Figure 7C) while most samples demonstrated weak to moderate cytoplasmic staining of the tumor cells, similar to profiles recently reported in melanoma and sarcoma [27, 46]. In most cases, perivascular staining was moderate to strong which is also in agreement with previous studies [19, 20]. Similar to the other tissue types examined, normal skeletal muscle demonstrated variable moderate staining of endothelial cells and strong perivascular cell staining, a finding that is consistent with those of Naylor, et al. [29].

## CONCLUSIONS

We have described the development and characterization of a panel of reagents, both monoclonal antibodies and recombinant proteins, that should prove useful for further elucidating endosialin/TEM-1 expression and biological function in cells/tissues from healthy and disease states, especially cancer. The mAbs described here recognize discrete extracellular subdomains within both the human and mouse endosialin/TEM-1 proteins and may aid in dissecting the roles of one or more domains in regulating endosialin/TEM-1 biological functions. The development of species cross-reactive mAbs enables their use for *in vivo* studies that were not possible with previously described mAbs that were species specific. Indeed, *in vivo* experiments aimed at further elucidating the biological functions of endosialin/TEM-1 with these diverse mAbs are ongoing.

Additionally, we have employed these reagents to develop immunoassays for the detection of endosialin/TEM-1 in fluids and tissues. Importantly, we have for the first time demonstrated the presence of a soluble form of endosialin/TEM-1, which we have termed sEND, in the serum and plasma of both healthy subjects and cancer patients. While the presence of sEND in systemic circulation does not appear different in the small sampling conducted in our studies, further studies of sEND in different cancer types and disease stage may find novel patterns that may be helpful for future patient selection and treatment criteria. The development of rodent/human cross reactive mAbs developed here may also enable the use of rodent models and human clinical specimens to aid in delineating the use of sEND and/or endosialin/TEM-1 as a predictive or prognostic biomarker for disease classification and/or therapeutic response. To this end, we have recently reported that endosialin/TEM-1 expression in soft tissue sarcomas (STS) as determined by IHC using mAb-9G5 is prognostic for a better outcome [46]. Follow on studies using these reagents are being designed to determine if there is a similar positive prognostic correlation with sEND in STS as well as in other cancer indications.

## MATERIALS AND METHODS

### Production of recombinant endosialin/TEM-1 fragments

For endosialin/TEM-1 cDNA constructs, as human endosialin/TEM-1 contains no introns, genomic DNA was purified from 2x10^6^ LA1-5S cells using the DNAEasy Kit (Qiagen, Hilden Germany) as per the manual. Genomic DNA was used for PCR amplification with primers homologous to the open reading frame (ORF) and including 5' *Hind*III and 3' *Xho*I restriction sites. The DNA fragment was double digested with *Hind*III and *Xho*I, gel purified, and ligated into pCEP4 plasmid digested in a similar manner. The construct was DNA sequenced to confirm integrity of the ORF. This construct served as a template for the subsequent subcloning of soluble fusion proteins of extracellular domains (ECDs). For TEM-1-Fc, the complete ECD (including lectin, sushi, EGF and mucin domains; LSEM) was cloned in-frame in an expression vector containing the murine IgG2b Fc domain mutated in the hinge region to produce monomeric TEM-1-Fc fusion protein. In a similar manner, the ECD was subcloned into a vector encoding a 6xHis C-terminal tag (TEM-1-6xHis). ECD fragments encoding only the lectin, Sushi, and EGF domains (TEM-1-LSE), the lectin and Sushi domains (TEM-1-LS), or the lectin domain (TEM-1-L) were generated in a similar manner. TEM-1-Fc and TEM-1-6xHis were stably transfected into CHO-K1 cells using lipofectamine 2000 (Invitrogen, Carlsbad, CA) as per the manual. TEM-1-LSE was transfected in a similar manner but was expressed in 293F cells.

For the expression of soluble TEM-1-L (Lectin domain of TEM-1, from 18Q to 163A) and TEM-1-LS (Lectin-Sushi domain of TEM-1, from Q18 to 234G) from insect cells, the DNA fragments for TEM-1-L and TEM-1-LS were generated by PCR using the primers designed to incorporate an N-terminal GP64 signal sequence and a C-terminal 6xHis and a thrombin cleavage sequence in front of the 8xHis tag into the translated protein. The resulting DNA fragments were cloned in the insect cell expression vector PIEx/Bac-1(EMD Millipore, Darmstadt, Germany). Recombinant baculovirus was produced using Bac Magic kit (EMD Millipore) following the manufacturer's instructions. TEM-1-L and TEM-1-LS were expressed from HighFive insect cells. 500 mLs of HighFive insect cell cultures (1.2-1.0x10^6^ cells/mL) were infected by recombinant baculovirus. Seventy-two hours post infection the medium was collected and TEM-1 fragments were purified

### Endosialin/TEM-1 fragment expression and purification

Chinese Hamster Ovary-K1 (CHO-K1) cells stably expressing the extracellular domain of human endosialin/TEM-1 as a single-chain mouse IgG2a Fc fusion protein were grown in 25L wave bags until viability was < 70%. Cell debris was removed by centrifugation and phenylmethylsulfonyl fluoride (PMSF) and EDTA were added to final concentrations of 0.1 mM and 5 mM, respectively. Harvested supernatant was passed through a 70 mL MORAb-004-Sepharose CL-4B affinity column. Following extensive washing with 1X PBS, 0.1 mM PMSF, pH 7.4 and 1X PBS, 2M NaCl, 0.1 mM PMSF, pH 7.4, bound material was eluted using 50 mM MOPS, 3M MgCl_2_, 0.1 mM PMSF, pH 6.8. Peak fractions were combined and dialyzed extensively against 1X PBS, 0.1 mM PMSF, pH 7.4. Yield was determined using BCA assay and purity was assessed by SDS-PAGE with Coomassie blue staining.

Hexa-histidine tagged human TEM-1-LSEM and TEM-1-LSE were expressed as secreted molecules in CHO-K1 or HEK293F cells, respectively. TEM-1-LS and TEM-1-L were expressed in HighFive insect cells. Recombinant proteins were purified from conditioned medium by immobilized metal affinity chromatography (NI-NTA, Qiagen). Purity was evaluated by SDS-PAGE analysis.

### Immunization, cell fusion and hybridoma cloning

Three eight-week old female Lewis rats were immunized with rhTEM-1-Fc fusion protein as follows: Initial intraperitoneal immunizations administered on day 0 comprised of 100 μg TEM-1-Fc prepared as a 1:1 (v:v) mix with complete Freund's adjuvant (Rockland Immunochemicals, Inc., Gilbertsville, PA). Boosts (50 μg immunogen mixed 1:1 (v:v) with incomplete Freund's adjuvant; Rockland Immunochemicals Inc.) were administered intraperitoneally on day 14 and every 21 days thereafter. Test bleeds were collected on day 24 and every 21 days thereafter and analyzed by direct enzyme-linked immunoassay (EIA) against TEM-1-6XHis. Spleens were harvested from animals exhibiting the highest antigen-specific titers and hybridomas were prepared by electrofusion (Hybrimune™ Model CEEF-50B Waveform Generator; Cellectis, Romainville, France) of splenocytes with Sp2/0 Ag14 myeloma cells (ATTC, Rockville, MD). Hybridoma supernatants were rescreened by EIA against rTEM-1-6XHis or recombinant mesothelin protein (Morphotek, Inc.) as a negative control, to identify hybridomas producing anti-rTEM-1 antibodies. Selected parental cell lines were then subcloned by limiting dilution and re-assayed against TEM-1-6XHis by EIA. Isotyping of selected clones was performed using the Clonetyping System (SouthernBiotech, Birmingham, AL).

### Primary monoclonal antibody (mAb) selection - Direct enzyme immunoassay (EIA)

mAb selection was performed using a direct enzyme immunoassay (EIA) technique. Briefly, 96-well plates were coated with 100 μL/well of a PBS solution containing 1 μg/mL TEM-1-6XHis overnight at 4°C. Wells were then washed and blocked with 3% (w/v) fish gel in PBS for 1 hr at RT. Next, 100 μL of a 3-fold dilution series of hybridoma culture supernatant was added to the wells and plates were incubated at RT for 1 hr. After washing 3 times with PBS containing 0.2% (v/v) Tween-20 (PBST), 100 μL of horseradish peroxidase- conjugated mouse anti-rat IgG (1:2500 dilution; Rockland Immunochemicals, Inc.) was added and plates were incubated for 30 min at 37°C. After washing with PBST, 100 μL of substrate (TMBE-100; Rockland Immunochemicals, Inc.) was added and the reaction stopped after 30 min by addition of 100 μL of 1M HCl. Plates were read at 450nm on a Benchmark microplate reader (BioRad, Concord, CA).

### mAb production and purification

Hybridoma cell lines were tested for mycoplasma using the mycoplasma PCR ELISA (Roche, Mannheim, Germany). Monoclonal antibodies were produced from mycoplasma negative cells grown in 1L roller bottle cultures. After seeding at 0.5x10^5^ cells/mL, cultures were grown for 21 days in medium (Invitrogen) with 5% low IgG fetal bovine serum (Gibco). At termination, culture supernatants were concentrated approximately 10-fold through a 50kDa filtration membrane (SpectrumLabs, Rancho Dominguez, CA) and mAbs were purified by Protein G affinity chromatography, eluted and subsequently dialyzed against PBS using a 12-14kDa membranous tubing (SpectrumLabs, Rancho Dominguez CA), sterile filtered using 0.22 μm Express™PLUS Stericups (Millipore, Billerica MA), aliquoted and stored at 4°C.

### Flow cytometry (FACS)

Aortic Smooth Muscle Cells (Invitrogen), HUVEC (ScienCell, Carlsbad, CA) cells, and SJSA-1 osteosarcoma cells (American Type Culture Collection (ATCC), Manassas, VA) were harvested using enzyme-free cell dissociation buffer (Invitrogen) or trypsin as indicated. Cells were washed twice in PBS and re-suspended at 5X10^4^ cells/100 uL FACS buffer (PBS+2% FBS) in a round bottom 96 well plate. Cells were incubated for 1hr on ice with primary antibodies at a concentration of 10 μg/mL, washed and then incubated with FITC-conjugated secondary antibodies (dilution 1:100) (Southern Biotech). Prior to analysis, cells were labeled with 7-AAD (BD Biosciences, Franklin Lakes, NJ) for the exclusion of nonviable cells. Cells were analyzed on an EasyCyte Flow Cytometer (Guava Technologies, Hayward, CA).

### Western blot analysis

Aortic smooth muscle cells and HUVECs were lysed in 1.1% OBG buffer (50 mM Tris-HCl, pH 7.5, 150mM NaCl, 1.1% OBG) supplemented with Complete Mini Protease Inhibitor Cocktail (Roche Diagnostics) plus PMSF (100 nM) for 15 min on ice, then centrifuged at 13,000 g for 15 min to remove debris. Equal amounts of total protein (30 μg) were added to NuPAGE sample buffer (Invitrogen) containing 5% β-mercaptoethanol plus 20 mM DTT. Proteins were separated using SDS-polyacrylamide gel electrophoresis (SDS-PAGE) on a 4-12 % Bis-Tris gel (Invitrogen) and transferred to PVDF membrane. Immunoblotting was conducted using Rockland antibodies overnight at 4°C, detected with a goat anti-rat-HRP conjugated antibody (Jackson Immunoresearch, West Grove, PA) and visualized using SuperSignal West Pico chemiluminescent substrate (Pierce). Signal was visualized using the Omega 12iC molecular imaging system (Ultralum, Biovision Technologies, Exton, PA).

### Mouse endosialin/TEM-1 cross-reactivity

Antibody cross-reactivity to mouse endosialin/TEM-1 was characterized using a direct EIA method. Briefly, human or mouse endosialin/TEM-1-6XHis (referred to as TEM-16XHis) were coated at 1 mg/mL overnight at 4°C onto 96 well plates. The plates were washed three times with PBST buffer (PBS buffer containing 0.05% Tween-20), then blocked with 5% BSA in PBST, at room temperature for 2 hr. The antibodies were serially diluted 1:5 starting at 5 mg/mL in PBST buffer containing 1% BSA, added to the plates, incubated for 2 hr at room temperature and then washed three times with PBST buffer. The goat-anti-Rat Ig(G+M)-HRP (Jackson Immunoresearch) was diluted 1:10,000 in PBST/1% BSA and incubated for 1 hr. The plates were washed again, developed with QuantaBlu Fluorogenic Substrate (ThermoFisher) and absorbance read at 325/420nm.

### Surface plasmon resonance (SPR)

SPR experiments were performed at 25°C using a Biacore T100 with research grade CM5 chips (GE Healthcare). Rat anti-TEM-1 mAbs were captured onto anti-mouse IgG-immobilized flow cells and MORAb-004 was captured onto an anti-human IgG-immobilized flow cell by injecting for 1 min at a flowrate of 20 μL/min.

A series of increasing concentrations of TEM-1-6XHis (1 nM, 3 nM, 10 nM, 30 nM, and 90 nM) was injected for 90 sec at a flowrate of 25 μL/min to record TEM-1-His binding. The dissociation of TEM-1-6XHis was monitored for 40 min at the same flowrate. TEM-1-6XHis was also injected into the anti-mouse IgG-immobilized flow cell and anti-human IgG-immobilized flow cell for base-line correction. To obtain a reference sensogram for each sample injection, HBS-P buffer was injected with the same method for TEM-1-6XHis. Sensograms were analyzed with Biacore T100 Evaluation Software using a 1:1 binding model.

### Patient samples

Serum and plasma samples were obtained from various commercial vendors with Institutional Review Board approvals and patient consent and were collected between 2009 and 2011 (Bioreclamation, Westbury, NY; Indivumed, Lewisburg, PA; Folio Biosciences, Columbus, OH; Discovery Life Sciences, Los Osos, CA). Tissue samples for immunohistochemistry were obtained from US Biomax (Rockville, MD).

### Immunoprecipitation of endosialin/TEM-1 from human serum

Serum from donors was pre-cleared with Protein G Sepharose FF Beads (Amersham). Cleared serum was collected by pelleting the beads by centrifugation. Immunoprecipitation was performed by adding MORAb-004 (2 μg) to the serum samples and incubating overnight, then washed Protein G Sepharose beads were added to the serum/antibody mixture and incubated for an additional 2 hr. After incubation, the samples were centrifuged and the supernatant was removed. The samples were then washed three times with cold lysis buffer 50 mM Tris-HCl, pH 7.5, 150 mM NaCl, 1.1% OBG supplemented with Complete Mini Protease Inhibitor Cocktail (Roche Diagnostics) plus PMSF (100 nM)). Protein was eluted using 4X NuPAGE sample buffer (Invitrogen) containing 5% ß-mercaptoethanol and boiled for 10 min. Proteins were separated using SDS-polyacrylamide gel electrophoresis (SDS-PAGE) on a 4-12 % Bis-Tris gel (Invitrogen) and transferred to PVDF membrane. Immunoblotting was conducted using a rabbit anti-TEM-1 antibody, detected with a goat anti-rabbit-HRP conjugated antibody (Jackson Immunoresearch) and visualized using SuperSignal West Pico chemiluminescent substrate (Pierce). Signal was visualized using the Omega 12iC molecular imaging system (Ultra-Lum).

### Soluble endosialin/TEM-1 electrochemilumin-escence (ECL) assay

The assay for detection of soluble endosialin/TEM-1 (sEND) was developed using the ECL technology from MesoScale Discovery. Ten purified mAbs were screened in a pairwise fashion against recombinant endosialin/TEM-1-Fc protein to identify potential capture and detection pairs. Selection of the final pair was based upon background, sensitivity, detection range, physical properties, and performance with clinical samples. mAb-9G5 was selected as the capture reagent and biotinylated. mAb-15F8 was selected as the detection mAb and labeled with ruthenium (SULFO-TAGTM NHS ester, MSD, Rockville, MD).

Samples (diluted 1:20 or 1:40), standards (4-fold dilutions starting at 5000 pg/mL), or controls (50 μL) were mixed with 50 μL of detection antibody and 50 μL of capture antibody in a polypropylene plate and incubated at room temperature for 60 min. Concurrently, STREPTAVIDIN GOLD plates were incubated with blocking reagent at room temperature with shaking (approximately 500-600 rpm) for 60 min. Following removal of blocker, 50 μL of the sandwich immunoassay component mixture was added to wells in duplicate and incubated with shaking for 60 min at room temperature. Plates were washed, read buffer added and signals measured on an MSD SECTOR imaging system.

### Immunohistochemistry

Endosialin/TEM-1 immunohistochemistry assay using mAb-9G5 was performed on FFPE samples sectioned at 4-5 μm and adhered to positively charged glass slides. Slides were deparaffinized and peroxidase blocked before being placed into heated Tris/EDTA buffer for a 20 min steamer pretreatment. After a 20 min cool down, the slides were rinsed in Tris-buffered saline with Tween 20 (TBST) and loaded onto Dako autostainers. UltraVision Protein Block was incubated for 5 min before a 40 min incubation with primary antibody. The slides were then rinsed with TBST, incubated with secondary antibody for 10 min, rinsed with TBST, incubated with HRP Polymer for 15 min, rinsed with TBST, incubated with 3, 3' diaminobenzidin (DAB) for 10 min, rinsed with TBST, counterstained with hematoxylin for 3 min, rinsed with TBST, dehydrated, and cover slipped. Tumor, stroma, vascular endothelial cells, and pericytes were scored by a board certified pathologist as percentages of target cells staining at the following intensities: 0 (defined as negative), 1+ (defined as weak), 2+ (defined as moderate), or 3+ (defined as strong).
